# P-2050. Ad26.COV2.S (Janssen) COVID-19 Vaccine Effectiveness against Hospitalization in Europe: an id.DRIVE Study

**DOI:** 10.1093/ofid/ofae631.2206

**Published:** 2025-01-29

**Authors:** Chloé Wyndham-Thomas, E Claire Newbern, Ainara Mira-Iglesias, Akshat Dwivedi, Alejandro Orrico-Sánchez, Andrés Antón, Charlotte Martin, Giancarlo Icardi, Irma Casas, Kok Yew Ngew, Laura Drikite, Leonie de Munter, Gerrit Luit ten Kate, Nikki Vroom, Sebastian Baumgartner, Susana Otero-Romero, Xavier Holemans, Kaatje Bollaerts, Nicolas Praet

**Affiliations:** P95 Epidemiology and Pharmacovigilance, Leuven, Vlaams-Brabant, Belgium; Janssen Research & Development, LLC, Horsham, Pennsylvania; Fisabio Public-Health, Valencia, Comunidad Valenciana, Spain; P95 Epidemiology and Pharmacovigilance, Leuven, Vlaams-Brabant, Belgium; FISABIO-Public Health, Valencia, Comunidad Valenciana, Spain; Hospital Universitario Vall d’Hebrón, Barcelona, Catalonia, Spain; Le Centre Hospitalier Universitaire St Pierre, Brussels, Brussels Hoofdstedelijk Gewest, Belgium; University of Genoa, San Martino Policlinico Hospital, Genoa, Liguria, Italy; Hospital Universitari Germans Trias i Pujol, Badalona, Catalonia, Spain; P95 Epidemiology and Pharmacovigilance, Leuven, Vlaams-Brabant, Belgium; P95 Epidemiology and Pharmacovigilance, Leuven, Vlaams-Brabant, Belgium; P95 Epidemiology and Pharmacovigilance, Leuven, Vlaams-Brabant, Belgium; Universitair Ziekenhuis Antwerpen, Edegem, Antwerpen, Belgium; P95 Epidemiology and Pharmacovigilance, Leuven, Vlaams-Brabant, Belgium; Klinik Favoriten, Wien, Wien, Austria; Hospital Universitario Vall d’Hebrón, Barcelona, Catalonia, Spain; Grand Hôpital de Charleroi, Charleroi, Hainaut, Belgium; P95 Epidemiology and Pharmacovigilance, Leuven, Vlaams-Brabant, Belgium; Janssen Research & Development, LLC, Horsham, Pennsylvania

## Abstract

**Background:**

Ad26.COV2.S (Janssen COVID-19 Vaccine) is a replication-incompetent viral-vector vaccine used in the international response to the SARS-CoV-2 pandemic. id.DRIVE (previously COVIDRIVE) is a public private partnership that conducts infectious disease studies on vaccines and therapeutics in the European region. Here we present a post-authorization study assessing the vaccine effectiveness (VE) of Ad26.COV2.S single dose against COVID-19 hospitalization.

**Methods:**

The study was non-interventional, multicentric, combined retrospective and prospective enrolment, and used a test-negative case-control design. Study participants were adults ≥ 18 years old, hospitalized with severe acute respiratory infection (SARI) between 1st May 2021 and 28th February 2023. All participants were tested for SARS-CoV-2 using RT-PCR or another RNA amplification system with equivalent diagnostic testing performances. VE against SARI hospitalization of Ad26.COV2.S single dose compared to unvaccinated was estimated as a single measure over the study period, and stratified by age group and different times since vaccination. All estimates were adjusted for symptom-onset date, age, sex, and number of chronic conditions among a predefined list of comorbidities that increase the risk of severe COVID-19 disease.

**Results:**

A total of 1318 SARI patients were included, 997 test-positive cases (COVID-19 cases) and 321 test-negative controls. Median age was 57 years, 584 (44%) were female and 785 (60%) had at least one chronic condition. Overall, VE was 55.6% (95% CI 23.6; 74.2) for a median of 146 days since vaccination. VE was 61.6% (95% CI 16.2; 82.4) for 18-49-year-olds, 57.7% (95% CI 3.4; 81.5) for 50-64-year-olds, and 40.8% (95% CI -6.0; 66.9) for ≥ 65-year-olds. By time since vaccination ≥ 14 days-to-12 weeks, 12-to-25 weeks, 25-to-52 weeks and > 52 weeks, VE was 52.4% (95% CI 2.8; 76.7), 59.2% (95% CI 25.0; 77.8), 35% (95% CI -50.2; 71.9) and 79.3 (95% CI -79.0; 97.6), respectively.

**Conclusion:**

Ad26.COV2.S single dose protected against COVID-19 hospitalization with VE lasting for at least six months. VE estimates were lower in older age groups. Results had low to medium levels of certainty and are to be interpreted with caution.

**Disclosures:**

Chloé Wyndham-Thomas, PhD, Janssen: This study was funded by Janssen E. Claire Newbern, PhD, Janssen: Employed by Janssen at the time of the study. Janssen funded this study.|Janssen: Stocks/Bonds (Private Company) Ainara Mira-Iglesias, PhD, Janssen: Janssen funded this study Alejandro Orrico-Sánchez, Janssen: Janssen partially funded the hospital network to collect data Irma Casas, PhD, Pfizer: Support from Pfizer to attend Congress in September 2023 Laura Drikite, MSc, Janssen: This study was funded by Janssen Nikki Vroom, MSc, Janssen: This study was funded by Janssen Sebastian Baumgartner, MD, GlaxoSmithKline: Lecture paid by GSK|MSD: Invitation to a meeting by MSD Susana Otero-Romero, PhD, GlaxoSmithKline: Honoraria|Pfizer: Support for attending meetings|Sanofi: Honoraria Kaatje Bollaerts, PhD, AstraZeneca: Advisor/Consultant|Bavarian-Nordic: Advisor/Consultant|CureVac: Advisor/Consultant|GlaxoSmithKline: Advisor/Consultant|Janssen: Advisor/Consultant|Novavax: Advisor/Consultant|P95: Stocks/Bonds (Private Company)|Pfizer: Advisor/Consultant|Valneva: Advisor/Consultant Nicolas Praet, PhD, Janssen: Employed by Janssen. Janssen funded this study.|Janssen: Stocks/Bonds (Private Company)

